# Artificial intelligence in refractive surgery: progress, challenges, and future directions

**DOI:** 10.3389/fcell.2026.1824307

**Published:** 2026-05-07

**Authors:** Yuke Ji, Lu Xie, Fangyan Liu, Yanwu Xu, Weihua Yang, Shaochong Zhang

**Affiliations:** 1 Shenzhen Eye Hospital, Jinan University, Shenzhen, Guangdong, China; 2 Shenzhen Eye Hospital, Shenzhen Eye Medical Center, Southern Medical University, Shenzhen, Guangdong, China; 3 School of Future Technology, South China University of Technology, Guangzhou, China

**Keywords:** artificial intelligence, corneal refractive surgery, deep learning, implantable collamer lens, machine learning, refractive surgery

## Abstract

The rapid evolution of artificial intelligence (AI) has catalyzed significant advancements in ophthalmology. As refractive surgery necessitates increasing levels of precision and personalization, AI offers pivotal solutions for optimizing clinical outcomes. This review systematically summarizes recent progress in applying machine learning and deep learning models to corneal refractive surgery and implantable collamer lens (ICL) procedures. Specifically, we examine AI’s utility in preoperative candidate screening, personalized surgical planning, and the prediction of postoperative complications. Although AI demonstrates broad prospects for enhancing surgical decision-making, several challenges remain, including data standardization, algorithm interpretability, cross-device compatibility, and ethical considerations. Overall, AI-driven decision-support systems are accelerating the transition of refractive surgery from standardized protocols to data-driven, individualized management, with the potential to enable more intelligent, automated, and precise surgical correction in ophthalmology.

## Introduction

Artificial Intelligence (AI), a pivotal branch of computer science, encompasses a range of technologies including Machine Learning (ML), Deep Learning (DL), and Computer Vision (CV) (Rahul, 2015; Bas et al., 2022; Janice et al., 2022). These technologies have demonstrated formidable capabilities in data processing and intelligent decision-making across various domains. In the medical field, AI has been extensively utilized for its proficiency in data analysis and recognition, particularly in medical imaging analysis, disease screening, intelligent diagnostics, and surgical planning (Xuxin et al., 2022; Demilade et al., 2022; Rabab Ali et al., 2024; Jordi et al., 2022). Recent advancements in big data technology, enhanced computational power, and breakthroughs in machine learning algorithms have significantly propelled the application of AI in medicine, markedly improving the accuracy of disease detection and the efficiency of diagnosis and treatment. In ophthalmology, AI technology is gradually becoming an important auxiliary tool for clinicians, especially in the early screening and treatment of common ocular diseases such as diabetic retinopathy (DR) (Wang et al., 2026; Khokhar et al., 2025), glaucoma (Angara et al., 2026; Mohammadzadeh et al., 2026), age-related macular degeneration (AMD) (Schmidt-Erfurth et al., 2025; Lee et al., 2026), keratitis (Erukulla et al., 2025; Prajna et al., 2025), and pterygium (Tiong et al., 2025; Ji et al., 2025). Leveraging machine learning and image recognition technologies, AI can efficiently analyze vast amounts of fundus images, enabling precise diagnosis. This not only enhances screening efficiency but also facilitates the early detection of subtle pathological changes, thereby optimizing therapeutic decision-making. As AI technology continues to mature, its value in clinical ophthalmology is expected to expand further, offering patients more accurate and efficient diagnostic and therapeutic solutions.

Refractive Surgery is a kind of surgery to correct refractive errors (such as myopia, hyperopia and astigmatism) by changing the optical characteristics of cornea or lens (Tae-Im et al., 2019; Beata and Alina, 2024). In recent years, the number of nearsighted people worldwide has surged, and the incidence of high myopia has increased significantly (Ian et al., 2017), which makes the demand for refractive surgery increase continuously. Refractive surgery is broadly classified into corneal refractive surgery and lens-based refractive surgery (Marcus et al., 2020). Corneal refractive surgery involves the precise laser ablation of corneal tissue to ensure proper focusing of light within the eye, thereby correcting refractive errors (Daizong et al., 2017). Lens-based refractive surgery is suitable for patients with high myopia or presbyopia who cannot be corrected by corneal refractive surgery (Andrzej et al., 2019). With advancements in medical technology, refractive surgery has evolved from standardized treatment protocols to personalized treatment plans, significantly enhancing the precision, safety, and postoperative visual quality of the procedures. The widespread application of refractive surgery has not only improved patients’ uncorrected visual acuity but also reduced their dependence on glasses and contact lenses, greatly enhancing their quality of life. Despite the continuous evolution of refractive surgery techniques, preoperative patient screening, surgical method selection, postoperative recovery, and long-term complication monitoring remain critical factors influencing surgical outcomes. The integration of AI into these areas offers novel solutions to these challenges. AI can optimize preoperative patient screening and improve the accuracy of preoperative assessments through the analysis of ocular data. Furthermore, AI can utilize image analysis to predict postoperative complications, thereby optimizing postoperative management strategies and enhancing the overall efficacy of refractive surgery.

Compared with previous reviews that mainly discussed the general applications of AI in ophthalmology or broadly summarized AI-assisted diagnosis in anterior segment diseases, the present review specifically focuses on the role of AI across the perioperative workflow of refractive surgery, including preoperative screening, surgical decision-making, parameter optimization, and postoperative outcome/complication prediction. We particularly emphasize corneal refractive surgery and implantable collamer lens (ICL) implantation because these two categories represent the major current refractive correction strategies in clinical practice, have distinct indications and risk profiles, and have also become the main scenarios in which AI has been most actively investigated. By concentrating on these two representative surgical pathways, this review aims to provide clinicians with a clearer understanding of where AI may support patient selection, individualized procedure planning, and risk stratification, while also offering researchers a structured overview of current evidence, unmet technical challenges, and future directions for model development, validation, and translation into refractive surgery practice.

## Methods

### Search strategy

To comprehensively identify studies related to the application of AI in refractive surgery, a systematic literature search was conducted in the PubMed database. Two distinct categories of search terms were defined to ensure a thorough retrieval of relevant studies. The search terms included: “Artificial Intelligence,” “Machine Learning,” “Deep Learning,” “Neural Networks,” “Refractive Surgery,” “Corneal Refractive Surgery,” and “ICL.” These terms were combined using “and” and “or” to create a comprehensive search strategy. Keywords from different categories were interchangeably used, thus covering a wide range of literature related to the research topic.

### Inclusion criteria

**FIGURE 1:**
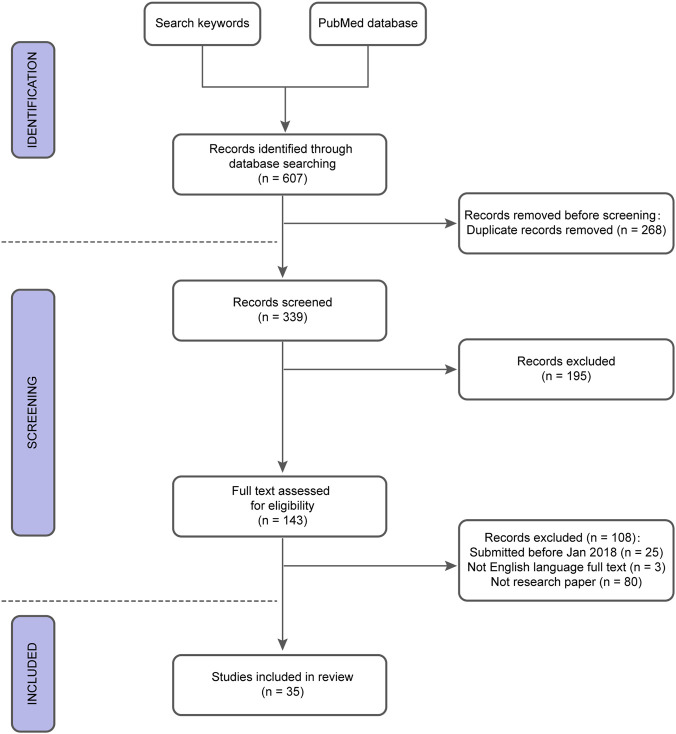
The flowchart of the study search.

To ensure the quality and relevance of the included studies, strict selection criteria were applied: (1) Publication Date: Studies published between January 2018 and June 2024 were included. (2) Language: Only studies published in English were considered. (3) Relevance: Studies must focus on the application of AI models or systems in refractive surgery. Any studies unrelated to this topic were excluded. (4) Study Type: Only original research articles were included. All not research papers were excluded, such as reviews, systematic reviews, meta-analyses, single case reports, and editorials. The study selection process is illustrated in Figure 1. Among the studies included in this review, priority was given to those utilizing high-quality datasets, large sample sizes, and external validation of AI models to ensure robustness and generalizability.

### AI and refractive surgery

#### Applications of AI in corneal refractive surgery

Corneal refractive surgery, a primary method for correcting refractive errors, encompasses various techniques such as Laser-Assisted *In Situ* Keratomileusis (LASIK), Photorefractive Keratectomy (PRK), and Small Incision Lenticule Extraction (SMILE) (Majid et al., 2021; Hassan et al., 2023). These surgeries aim to correct refractive errors by altering the curvature or thickness of the cornea, thereby optimizing light focusing. The success of corneal refractive surgery depends not only on the choice of surgical technique but also on preoperative assessments, personalized surgical planning, intraoperative precision, and postoperative management. However, due to variations in individual anatomical structures and physiological responses, complications such as reduced visual quality, refractive regression, dry eye syndrome, and corneal ectasia may still occur postoperatively (FangJun et al., 2022; Ane and Pilar, 2017; Debora et al., 2014; Sridevi et al., 2023). Consequently, improving the precision, personalization, and postoperative stability of corneal refractive surgery remains a critical research focus in this field. In recent years, AI technologies, particularly ML and DL, have demonstrated significant potential in medical image analysis and surgical planning. In corneal refractive surgery, AI can be trained on large-scale clinical datasets to assist clinicians in screening suitable surgical candidates, selecting appropriate surgical techniques, predicting postoperative visual outcomes, and assessing the risk of complications. These advancements are driving refractive surgery toward greater precision and personalization, ultimately enhancing patient outcomes.

#### Applications of AI in preoperative care for corneal refractive surgery

Although corneal refractive surgery is highly effective in correcting refractive errors, not all patients are suitable candidates. Inappropriate patient selection can lead to corneal complications, postoperative visual deterioration, or even irreversible corneal damage (Kraig and Fasika, 2014; O-U et al., 2022). Therefore, rigorous preoperative screening to identify suitable candidates is critical for ensuring surgical safety and long-term stability. Currently, the selection of candidates for corneal refractive surgery primarily relies on clinicians’ experience, which is not only susceptible to individual variability but also time-consuming, potentially compromising diagnostic efficiency. To address these challenges, Tae et al. (2019) investigated the application of ML in screening candidates for corneal refractive surgery. They developed an automated candidate identification system using multiple ML algorithms and ensemble learning methods, combined with large-scale clinical data. The results demonstrated that ML models outperformed traditional methods in preoperative screening, with the ensemble model achieving the best performance (AUC = 0.972 in an external validation dataset) and showing robust predictive capabilities for high-risk patients (high myopia, high astigmatism, or thin corneas). Similarly, Yi et al. (2020) developed and validated a DL model based on corneal tomography images for automated candidate screening. The model utilized the InceptionResNetV2 convolutional neural network and was trained and tested on 6,565 corneal tomography images. Finally, the model achieved an accuracy of 0.95 on an independent test dataset, performing comparably to experienced refractive surgeons. These studies highlight the significant potential of AI in preoperative candidate screening, demonstrating its ability to improve screening accuracy and reduce the time required for manual screening, thereby enhancing clinical efficiency.

Corneal refractive surgery encompasses a variety of surgical methods, each with specific indications, advantages, and limitations (Renato and Steven, 2003). Different surgical methods vary significantly in terms of postoperative visual recovery speed, visual quality, intraoperative trauma, and risk of complications. Selecting the most appropriate surgical methods is crucial not only for achieving optimal postoperative visual outcomes but also for minimizing the risk of complications and ensuring long-term visual stability. To address this, Tae et al. (2020) developed an interpretable AI prediction system using multiple ML algorithms to recommend the most suitable refractive surgery method based on clinical data. The study utilized ocular data from 18,480 refractive surgery patients for model training and validation. External validation results showed that the AI system achieved a recommendation accuracy of 0.789, demonstrating its potential in refractive surgery planning. Similarly, Jiajing et al. (2024) constructed a series of AI prediction models using multiple ML algorithms to evaluate the ability of AI in assisting clinicians with surgical method selection. The study collected ocular examination data from 7,081 patients for model training and testing, employing small number of oversampling techniques to balance data distribution and reduce the impact of class imbalance on model performance. Among the tested models, the Random Forest model exhibited the best performance, achieving an accuracy of 0.8775 and demonstrating high consistency with clinical decisions made by ophthalmologists. These findings further validate the value of AI in assisting surgeons with surgical planning. By integrating clinical big data with AI technology, these models can provide personalized surgical recommendations, reduce subjectivity in decision-making, and enhance the scientific consistency of surgical planning, offering new possibilities for individualized treatment in refractive surgery.

Overall, these studies share the common goal of improving preoperative decision-making for corneal refractive surgery, particularly in candidate screening and procedure selection. Most studies demonstrated that AI models can achieve good predictive performance and show substantial agreement with expert clinical judgment. However, differences remain in the types of input data used, ranging from routine ophthalmic examination parameters to corneal tomography images, as well as in model architectures, including conventional machine learning algorithms, ensemble methods, and deep learning networks. These differences suggest that AI can be flexibly adapted to different clinical scenarios, while also highlighting the need for standardized evaluation and broader external validation before widespread implementation.

#### Applications of AI in postoperative care of corneal refractive surgery

Postoperative myopic regression is one of the most common long-term complications of refractive surgery (Kazutaka et al., 2007), which not only affects patients’ visual quality but also imposes additional economic burdens. There are many factors leading to postoperative myopia regression, such as the patient’s age, preoperative diopter and dry eye (Jihong et al., 2019; Mabel et al., 2018). Accurately identifying high-risk patients prone to myopic regression is crucial for developing personalized treatment plans and optimizing surgical outcomes. Juntae et al. (2022) developed an AI system based on the XGBoost and ResNet50 models, integrating preoperative clinical data and fundus images to predict the risk of myopic regression after corneal refractive surgery. The results demonstrated that the system performed well in predicting postoperative myopic regression (≥0.5D), achieving an AUC of 0.753. This study validated that AI models combined with fundus imaging can effectively improve the accuracy of preoperative risk assessment for myopic regression, thereby aiding in the selection of personalized surgical plans. SMILE, a minimally invasive refractive surgery, is known for its safety and stable corrective outcomes. However, postoperative overcorrection or undercorrection remains a significant factor affecting visual quality (Hong-Ying et al., 2017). To optimize postoperative visual acuity, precise adjustment of surgical parameters is essential, as it not only improves surgical success rates but also reduces the risk of postoperative refractive errors. Addressing this issue, Tong et al. (2019) developed a preoperative surgical parameter prediction model for SMILE based on an artificial neural network (ANN) and evaluated its potential to enhance postoperative predictability and reduce refractive errors. The research team trained and validated the model using surgical records from 1,746 eyes and compared its predictions with parameters set by surgeons. The results showed that the model matched surgeons in safety while significantly outperforming them in postoperative efficacy and predictive accuracy. This indicates that ANN-based prediction models can improve the accuracy of postoperative refractive state prediction in SMILE, reducing the risk of overcorrection or undercorrection. Similarly, Seungbin et al. (2021) constructed a series of AI models using multiple ML algorithms to predict SMILE surgical parameters. These models were trained and validated using examination data from 3,034 eyes. The results demonstrated that the AdaBoost model performed best, achieving accuracies of 0.969, 0.976, and 0.994 in predicting sphericity, cylinder, and astigmatism axis (with errors ≤0.25D or ≤25°), respectively. In another study, Mingdong et al. (2024) developed a ML model to analyze the impact of preoperative and intraoperative parameters on postoperative refractive outcomes after SMILE. The results revealed that corneal biomechanical properties, surgical parameters, and preoperative refractive status significantly influenced postoperative refractive outcomes. Key predictive factors included the percentage of corneal ablation thickness, maximum lenticule thickness, residual stromal thickness, and corneal stress-strain index. These findings highlight the importance of preoperative parameter adjustment in SMILE, help optimize preoperative surgical planning. Collectively, these studies show that AI technology can effectively improve the prediction accuracy of postoperative refractive state, reduce the occurrence of postoperative overcorrection or undercorrection, and provide patients with safer and more efficient visual correction solutions. A summary of these studies is presented in Table 1.

**TABLE 1:** Summary of AI application in corneal refractive surgery.

Year	Authors	Type of data	Sample size	AI algorithm	Performance
2019	Tae et al. (2019)	Ophthalmic examination data	18480 patients	ML	AUC = 0.972
2020	Yi et al. (2020)	Corneal tomography images	6565 images	DL	ACC = 0.95
2020	Tae et al. (2020)	Ophthalmic examination data	18480 patients	ML	ACC = 0.789
2024	Jiajing et al., 2024)	Ophthalmic examination data	7081 patients	ML	ACC = 0.8775
2022	Juntae et al. (2022)	Ophthalmic examination data	2009 eyes	ML	AUC = 0.753
2019	Tong et al. (2019)	Surgery records	1746 eyes	Artificial neural network	The model matched surgeons in safety while significantly outperforming them in postoperative efficacy and predictive accuracy
2021	Seungbin et al. (2021)	Ophthalmic examination data	3034 eyes	ML	The accuracy of sphericity, cylinder, and astigmatism axis (error ≤0.25D or ≤25°) was 0.969, 0.976, and 0.994, respectively
2024	Mingdong et al. (2024)	Clinical parameters	922 eyes	ML	The AI model could help the design of preoperative surgical plan

Abbreviations: ML, machine learning; DL, deep learning; ACC, accuracy; AUC, area under the curve.

In summary, the studies on postoperative care of corneal refractive surgery consistently indicate that AI has value in predicting refractive outcomes, myopic regression, and the need for individualized parameter adjustment. Their common strength lies in the ability to integrate multidimensional preoperative and surgical data to improve postoperative predictability. At the same time, these studies differ in their prediction targets, including regression risk, nomogram refinement, and postoperative refractive status, as well as in the clinical variables emphasized by different models. These findings suggest that AI may support more precise and personalized postoperative management, although further validation across different surgical platforms and patient populations remains necessary.

The aforementioned studies demonstrate that the application of AI is driving corneal refractive surgery toward greater precision, personalization, and intelligence. The integration of ML models has significantly enhanced preoperative screening, selection of surgical methods, preoperative parameter optimization, and postoperative complication prediction. As AI technology continues to mature, its applications in corneal refractive surgery are becoming increasingly sophisticated. This progress holds the potential to further refine surgical workflows, improve postoperative visual outcomes, and reduce the incidence of complications. Ultimately, these advancements will provide patients with safer, more efficient, and highly personalized vision correction solutions.

#### Applications of AI in implantable collamer lens (ICL) surgery

Implantable Collamer Lens (ICL) surgery is a pivotal refractive procedure for correcting high myopia, hyperopia, and astigmatism (Xun et al., 2021). It is particularly suitable for patients who are ineligible for corneal laser surgeries, such as LASIK or PRK, due to insufficient corneal thickness or other contraindications (Donald et al., 2004). Compared to corneal refractive surgeries, ICL implantation offers distinct advantages, including reversibility, unchanged corneal tissue, and a broader range of applications (Carlos et al., 2015), making it an increasingly popular choice among patients. The success of ICL surgery hinges on the stability of the implanted lens within the anterior or posterior chamber and its compatibility with intraocular structures. Vault control, ICL size selection and accurate preoperative evaluation are the key factors that affect the success of the operation (Jad et al., 2024; Jian et al., 2024). These factors directly determine postoperative visual quality, safety, and the likelihood of complications. Consequently, enhancing the precision and personalization of ICL surgery has become a critical focus in refractive surgery research. With the continuous advancement of AI technology, its applications in ICL surgery are increasingly deepening. AI can leverage large-scale clinical data to develop intelligent predictive models capable of accurately calculating the ideal vault range, determining the optimal ICL size, and predicting postoperative complication risks. These capabilities enable clinicians to design more scientific and personalized surgical plans, ultimately improving patient outcomes.

#### Applications of AI in preoperative care for ICL surgery

The selection of an appropriate ICL size is one of the most critical factors determining the success of ICL surgery. An optimal ICL size not only ensures the stability of the lens within the ciliary sulcus but also directly influences vault control, aqueous humor circulation, and the incidence of postoperative complications. An oversized ICL may lead to excessive vault, resulting in elevated intraocular pressure and secondary glaucoma (Paulo et al., 2011; Raccurt et al., 1991), while an undersized ICL may cause lens instability, increasing the risk of cataract formation (José et al., 2010). Therefore, precise ICL size selection is essential for enhancing surgical safety and efficacy. To address this challenge, Kang et al. (Eun et al., 2021) developed an ensemble AI model using XGBoost and Light Gradient Boosting Machine (LightGBM) to predict the optimal ICL size. After training and validation, the model demonstrated superior accuracy in ICL size prediction compared to other ML models and traditional sizing methods, offering a novel approach for ICL size selection. Similarly, Yang et al. (Yixuan et al., 2024) constructed a clinical decision support system based on convolutional neural networks (CNNs) to optimize ICL size selection by predicting postoperative vault. The system was trained and tested using preoperative measurement data from 2,772 eyes, achieving a prediction accuracy of 0.9137. This significantly improved the precision of ICL size selection, providing clinicians with a reliable preoperative reference. In another study, Heng et al. (2023) developed an innovative AI model using a data-level data-balancing approach combined with ML algorithms to predict the optimal ICL size. The model was trained and tested on preoperative measurement data from 1,127 eyes. The results demonstrated that the data-balancing approach not only addressed data imbalance issues but also significantly enhanced the model’s predictive performance. Compared to traditional linear regression models, this AI model exhibited higher accuracy in ICL size prediction, offering robust support for precise ICL surgery. Furthermore, to enhance the predictive capabilities of AI models, Jun et al. (2024) employed a multi-algorithm integration strategy to construct a majority-vote model. By combining the strengths of multiple ML algorithms, the model achieved excellent performance in ICL size prediction, with an accuracy of 0.853 and an AUC value of 0.973. These findings highlight that multi-algorithm integration strategies can not only improve model performance but also significantly enhance prediction accuracy, providing new insights for personalized and precise ICL surgery.

Taken together, the studies in this section mainly focus on optimizing preoperative ICL sizing and improving the accuracy of vault-related planning. A common finding is that AI-based models generally outperform or complement traditional nomograms and empirical methods in identifying the most appropriate lens size. Nevertheless, the included studies differ in sample size, feature selection, balancing strategies, and algorithm design, reflecting the complexity of ICL sizing in different clinical settings. Overall, these studies support the potential of AI to enhance preoperative precision in ICL surgery, while also underscoring the importance of multicenter validation and cross-device applicability.

#### Applications of AI in postoperative care of ICL surgery

Vault, defined as the distance between the implanted ICL and the anterior surface of the crystalline lens, is a critical parameter for assessing the proper positioning of the ICL (Alfonso et al., 2012). An appropriate vault ensures the stable placement of the ICL within the ciliary sulcus while preventing abnormal contact with key intraocular structures such as the iris, lens, and cornea. Excessive or insufficient vault can lead to severe complications, including elevated intraocular pressure, secondary glaucoma, lens opacities, and ICL dislocation (Paulo et al., 2011; Jae et al., 2019; M Hermina et al., 2020). Therefore, accurate prediction of postoperative vault is essential for ensuring the safety and stability of ICL surgery. To address this challenge, Nasser et al. (Taj et al., 2024) developed an AI model using deep learning algorithms to predict postoperative vault following ICL implantation. The model was trained and tested on 3,059 digital ultrasound images of the eye. The results demonstrated that the AI model achieved high accuracy in predicting postoperative vault, with most predictions falling within clinically acceptable ranges, providing robust support for ICL surgery. Similarly, Assaf et al. (Jad et al., 2023) developed a DL-based AI model using anterior segment optical coherence tomography (AS-OCT) to precisely predict postoperative vault. The model was trained and validated using biological data obtained from AS-OCT, and its predictive performance was evaluated using mean absolute error (MAE) and mean absolute percentage error (MAPE). The results showed that the model achieved an MAE of 15.82 µm and an MAPE of 3.42%, indicating the DL model could be used as an auxiliary tool in clinical practice. To further optimize the accuracy of postoperative vault prediction, Kamiya et al. (Kazutaka et al., 2021) constructed an AI model based on AS-OCT-derived biometric data and multiple ML algorithms, comparing the predictive performance of different algorithms. The model was trained and tested using AS-OCT images from 1,745 eyes. The results revealed that the random forest regressor model exhibited the lowest MAE, demonstrating the best predictive performance. In another study, Di et al. (Yu et al., 2024) compared the performance of 3 ML algorithms in predicting postoperative vault after ICL implantation and evaluated their predictions against the manufacturer’s nomogram. The results indicated that XGBoost outperformed other algorithms in both predictive accuracy and error control, highlighting the high applicability of ML models in clinical vault prediction. Russo et al. (Andrea et al., 2023) developed an AI model using ML algorithms to predict postoperative vault. Compared to the traditional manufacturer’s nomogram, the AI model demonstrated higher accuracy, providing clinicians with more precise preoperative vault assessments and optimizing ICL implantation plans to enhance surgical safety and efficacy. In another study, the research team (Xi et al., 2023) developed an AI model capable of adapting to different ophthalmic examination devices or combinations of devices. They integrated data from multiple diagnostic devices to train and test the AI model. This model not only achieved high accuracy in postoperative vault prediction but also demonstrated superior adaptability to cross-device data compared to previous studies. Compared with traditional methods, this model can be reliably applied across different medical institutions, offering robust vault prediction support for diverse clinical settings.

Although ICL surgery has demonstrated excellent clinical outcomes in correcting high myopia, hyperopia, and astigmatism, and has become a vital option for patients who are ineligible for corneal refractive surgery, complications such as secondary glaucoma and refractive errors may still occur in some patients. These complications are often attributable to variations in individual anatomical structures, ICL size selection, and postoperative ocular adaptation. These complications not only affect postoperative visual quality but may also have long-term adverse effects on ocular health. Therefore, accurately predicting postoperative complications and enabling early intervention are crucial for enhancing the long-term safety and efficacy of ICL surgery. Postoperative changes in anterior chamber anatomy, such as angle narrowing and elevated intraocular pressure, can lead to secondary glaucoma (Xun et al., 2021). Among these, reduced anterior chamber angle opening is considered a significant risk factor for postoperative glaucoma. Studies have shown that postoperative changes in the anterior chamber angle are closely related to ICL size and postoperative vault (Sherif et al., 2017). To predict these changes, Choi et al. (Hannuy et al., 2023) developed an ML-based predictive model. Through training and testing, the model demonstrated the ability to accurately predict postoperative anterior chamber angle changes, enabling clinicians to better assess postoperative angle status, identify potential risks of secondary glaucoma, and implement targeted interventions. In addition to predicting postoperative glaucoma risk, AI has shown promising applications in predicting postoperative refractive errors and calculating ICL power. Jiang et al. (Yinjie et al., 2023) developed a novel stacking ML model based on ML algorithms to predict postoperative refractive status and calculate ICL power. The model was trained and validated using extensive clinical data, and the results demonstrated its superior accuracy in predicting postoperative spherical equivalent (SE) and spherical power compared to traditional empirical formulas. This study highlights the advantages of ML in predicting postoperative refractive errors and enabling personalized ICL power calculation, with the potential to further enhance the precision and customization of ICL surgery, ultimately optimizing postoperative visual outcomes. A summary of these studies is presented in Table 2.

**TABLE 2:** Summary of AI application in ICL implantation.

Year	Authors	Type of data	Sample size	AI algorithm	Performance
2021	Eun et al. (2021)	Pre-operative ocular examination data	3739 eyes	ML	ACC = 0.759 ACC = 0.674
2024	Yixuan et al. (2024)	Ophthalmic examination data	2772 eyes	Conventional neural networks	ACC = 0.9137
2024	Heng et al. (2023)	Pre-operative ocular examination data	1127 eyes	ML	The AI model had higher accuracy in predicting ICL size
2024	Jun et al. (2024)	ocular examination data	1763 eyes	ML	ACC = 0.853 AUC = 0.973
2023	Taj et al. (2024)	Digital ultrasound images	3059 images	DL	The AI model had high accuracy in predicting postoperative vaults of ICL
2023	Jad et al. (2023)	AS-OCT images	2647 images	DL	The average absolute error is 15.82 µm and the average absolute percentage is 3.42%
2023	Kazutaka et al. (2021)	AS-OCT images	1745 eyes	SVR; GBR; RFR; LR	The random forest regressor model has the lowest mean absolute error in predicting vault
2024	Yu et al. (2024)	Images	707 eyes	ML	The XGBoost had the best prediction performance
2022	Andrea et al. (2023)	AS-OCT images	561eyes	ML	Compared to traditional manufacturer nomograms, the AI model had higher predictive accuracy
2023	Xi et al. (2023)	Image	1949 eyes	ML	The AI models could accurately predict the vault after ICL surgery
2023	Hannuy et al. (2023)	AS-OCT images	550 eyes	ML	The model could accurately predict the postoperative anterior chamber angle
2023	Yinjie et al. (2023)	Pre-operative ocular examination data	2767 eyes	ML	The model had superior predictive performance compared to traditional empirical formula-based calculation methods

Abbreviations: ML, machine learning; DL, deep learning; ACC, accuracy; AUC, area under the curve; SVR, port vector regressor; GBR, gradient boost regressor; RFR, random forest regressor; LR, linear regressor; AS-OCT; anterior segment optical coherence tomography.

Overall, studies on postoperative AI applications in ICL surgery consistently emphasize vault prediction as a central issue, while also extending to anterior chamber angle changes, refractive outcomes, and ICL power calculation. Their shared conclusion is that AI can improve the accuracy and individualization of postoperative assessment compared with conventional formulas or manufacturer nomograms. Meanwhile, these studies differ in imaging modalities, biometric inputs, and predictive endpoints, including AS-OCT-based vault estimation, ultrasound-based analysis, and postoperative angle or refractive prediction. These differences highlight both the versatility of AI approaches and the need for more unified validation standards to support broader clinical adoption.

In conclusion, ICL surgery is a safe and effective refractive correction method, but its outcomes are influenced by multiple factors, with vault and ICL size selection being particularly critical. An appropriate vault ensures the stability of the ICL within the eye, preventing complications such as lens opacities and elevated intraocular pressure, while precise ICL size selection optimizes postoperative visual quality and enhances surgical safety. The aforementioned AI research demonstrates that AI models are driving ICL implantation toward greater precision, efficiency, and personalization. As AI technology continues to mature, its applications in ICL surgery hold immense potential to further refine surgical workflows, improve postoperative visual outcomes, and reduce complications, making ICL implantation safer and more effective. These advancements are poised to bring new breakthroughs in refractive correction surgery.

### Limitations and challenges

Although AI has demonstrated significant potential and unique advantages in the field of refractive surgery, most AI systems or models remain in the experimental stage. This is not only due to the ongoing development of AI technology but also because of the numerous limitations and challenges encountered during its clinical transformation. Below, we summarize the primary limitations and challenges faced by AI in this field.

1. Data Quality and Diversity (Daniel et al., 2018). The training of AI models relies on large-scale, high-quality datasets to ensure robust decision-making capabilities. However, data related to refractive surgery may be limited by factors such as region, equipment, and patients, which can affect the generalizability of AI models. Furthermore, if an AI model is primarily trained on data from a specific population, its applicability to other populations may be significantly reduced. Therefore, ensuring data diversity and improving data quality are among the core challenges in advancing the application of AI in refractive surgery.
2. Dataset Construction and Annotation Standardization (Daniel et al., 2020). High-quality AI models depend on accurately labeled training data, and the construction and annotation quality of datasets directly influence model performance. Currently, the annotation of medical imaging data primarily relies on clinicians, which not only consumes significant time and human resources but also makes the creation of high-quality, standardized datasets extremely challenging. Therefore, establishing unified annotation standards and efficient annotation methods is a critical issue for the application of AI in refractive surgery.
3. Impact of Individual Variability (Yuke et al., 2023). Refractive surgery is highly personalized, as each patient exhibits unique corneal morphology, biomechanical properties, postoperative recovery capabilities, and visual demands. Such individual variability poses significant challenges for AI in predicting surgical outcomes. Additionally, postoperative recovery is influenced by multiple factors, many of which are difficult to fully incorporate into AI training datasets. Therefore, enabling AI to more accurately adapt to individualized needs and provide tailored surgical plans is a critical issue that needs to be solved in current research.
4. Interpretability of AI Algorithms (Dawei et al., 2023). While many AI models have demonstrated high accuracy in predicting refractive surgery outcomes and selecting surgical methods, their lack of transparency often results in poor interpretability. This directly reduces clinicians’ trust in AI and hinders its acceptance in clinical practice. Therefore, improving the interpretability of AI models to provide clear decision-making rationale is a crucial direction for advancing the clinical translation of AI.
5. Patient Privacy and Data Security (Blake, 2021). AI systems require access to and processing of large amounts of patient data, which often include sensitive information. Insufficient security measures during data collection, storage, or transmission can lead to privacy breaches or data misuse. Balancing data utilization with robust security measures remains a significant ethical and legal challenge in AI applications. Establishing stringent data management protocols is essential for ensuring the safe application of AI in refractive surgery.
6. Compatibility with Different Devices (Nicola et al., 2022). Refractive surgery relies on a variety of high-precision ophthalmic equipment, such as corneal topography, anterior segment optical coherence tomography (OCT), and excimer laser systems. These devices, often from different manufacturers, may exhibit significant variations in measurement methods, data formats, and parameter standards. Such inconsistencies limit the adaptability of AI models across different hospitals or devices. Therefore, establishing unified data format standards and enhancing the compatibility of AI models with diverse equipment data are critical steps toward enabling cross-hospital, cross-regional, and cross-device AI applications.
7. Legal Liability and Responsibility Attribution (Smith et al., 2024). With the increasing use of AI in refractive surgery planning and decision support, legal liability has become an important issue in clinical practice. If an AI-generated recommendation, such as ICL size selection or postoperative risk assessment, contributes to an inappropriate surgical plan and leads to complications, the attribution of responsibility remains unclear. In current clinical settings, AI is mainly used as an auxiliary tool, and the final treatment decision is still made by the physician. Nevertheless, as AI systems become more deeply integrated into clinical workflows, the boundaries of responsibility among clinicians, medical institutions, and algorithm developers may become more complex. Therefore, establishing clear legal and regulatory frameworks, defining the role of AI in clinical decision-making, and ensuring the traceability of AI outputs are essential for the safe and responsible application of AI in refractive surgery.

## Future perspectives

Overall, the application of AI technology in the field of refractive surgery has made significant progress, with far-reaching impacts on improving surgical accuracy, formulating personalized surgical plans, and predicting postoperative complications. With the rapid development of big data and AI algorithms, the application of AI in refractive surgery will continue to deepen, advancing toward greater precision and intelligence. In the future, the application of AI will be primarily reflected in the following aspects: (1) AI-Driven Preoperative Screening and Candidate Selection. In the future, with the accumulation of more high-quality clinical data, AI screening models will be continuously optimized. By integrating diverse examination data, such as corneal topography and corneal thickness, AI will form a more intelligent screening system. AI can not only improve screening accuracy but also significantly enhance diagnostic efficiency, enabling clinicians to identify high-risk patients more quickly and provide them with personalized treatment recommendations. As AI continues to advance in the field of medical image recognition, fully automated preoperative screening is expected to become a reality. This will allow clinicians to obtain comprehensive patient evaluation reports more efficiently, thereby optimizing clinical decision-making and improving the convenience and accuracy of screening. (2) AI-Assisted Surgical Planning and Individualized Treatment. In the future, AI will not only assist clinicians in surgical planning but also enhance the accuracy of surgical recommendations through continuous training and optimization. In complex cases, AI may even provide multiple surgical options. By learning the relationship between extensive preoperative examination data and postoperative refractive outcomes, AI models can accurately predict optimal surgical parameters. Furthermore, AI is expected to refine the calculation methods for surgical parameters and integrate real-time image analysis technology, enabling dynamic adjustments to surgical strategies during the procedure. This will further improve the precision and personalization of refractive surgery. Such intelligent surgical planning approaches will enhance postoperative visual quality and reduce the incidence of complications. (3) Application of AI in Predicting Postoperative Complications. The application of AI technology in postoperative management of refractive surgery is progressively deepening, offering new methods to improve postoperative safety and reduce complication rates. With the continued advancement of AI in predicting postoperative complications, it is anticipated that a more intelligent and individualized postoperative management system will be developed. By leveraging multimodal data fusion, big data analytics, and AI-based prediction models, this system will provide comprehensive postoperative management for patients. (4) Combining AI and Surgical Robots to Promote Refractive Surgery Automation. With the continuous advancement of AI technology, the integration of AI and surgical robots holds broad prospects in the field of refractive surgery. An automated refractive surgery system powered by AI technology will significantly enhance the stability and reproducibility of surgical procedures, reduce subjective errors in surgeons’ operations, and ultimately achieve more precise and safer visual correction outcomes. In the future, a fully automated refractive surgery platform that combines AI and robotics is expected to streamline the surgical process, improving efficiency and providing technical support for remote surgery. This will enable high-quality refractive surgery services to transcend geographical limitations, benefiting a larger number of patients. (5) Integration into Real-World Clinical Workflows and Primary Care. Integration into Real-World Clinical Workflows and Primary Care. In real-world clinical practice, AI models in refractive surgery are more likely to function as decision-support tools embedded within existing workflows rather than as replacements for clinicians. They may serve as initial screening tools to identify high-risk patients or those unsuitable for specific procedures, thereby improving efficiency and reducing the burden of manual assessment. In surgical planning, AI can offer additional guidance for procedure selection, parameter optimization, and complication risk prediction, while final decisions should remain the responsibility of the treating ophthalmologist. In the future, these models may be especially useful in primary-level hospitals and resource-limited settings, where they could help improve the consistency and quality of refractive surgery-related evaluations. Furthermore, AI systems with robust cross-device adaptability and external validation may support multicenter collaboration by promoting more standardized and efficient decision-making across institutions.

## Conclusion

In summary, the application of AI technology is transforming every aspect of refractive surgery, from preoperative screening and surgical method selection to postoperative complication prediction. AI is progressively enhancing the accuracy and personalization of refractive surgery. ML and DL-based AI models can efficiently analyze ocular imaging and clinical data, providing valuable assistance in candidate screening, surgical method selection, parameter optimization, and complication prediction, thereby improving diagnostic efficiency and surgical safety. Simultaneously, advancements in AI technology are driving the transition of refractive surgery from standardized to personalized approaches, offering patients more precise and effective treatment solutions. However, the clinical translation of AI in refractive surgery still faces challenges related to data quality, model interpretability, device compatibility, and ethical safety. In the future, with the accumulation of big data, continuous optimization of AI algorithms, the application of AI in refractive surgery will become more profound. AI has the potential to enable intelligent and automated surgical procedures, providing safer, more accurate, and more efficient correction solutions for patients with refractive errors. These advancements will further propel the development of refractive surgery, ushering in a new era of precision medicine in ophthalmology.
